# Literary Notes

**Published:** 1898-04

**Authors:** 


### Literary Notes.

Dr. I. N. Love, editor of the *Medical Mirror*, has again become its sole owner and publisher. Dr. Love has secured the stock in the *Mirror* lately owned by Mr. C. R. H. Davis, and has associated with him Dr. H. R. Hall, a native of St. Louis, a graduate of the Washington University, of the Missouri Medical College and the City Hospital as assistant editor, who will be so thoroughly identified with the work as to be a very important factor. Dr. Hall recently served a term as assistant city physician and has such a fund of experience and general culture as to bespeak for him a pronounced success in practice and medical journalism.

We congratulate Dr. Love upon again securing absolute control of his own and bespeak for the *Mirror* a continuously increasing prosperity.

The *Southwestern Medical and Surgical Reporter*, of Fort Worth, and the *Texas Medical Practitioner*, of Dallas, have been absorbed by the *Texas Medical News*. The *News* has increased its size by sixteen pages and presents evidences of prosperous improvement, in which it has our best wishes.

*Practical Therapeutics* is the name of a little periodical published by H. K. Mulford Company, Philadelphia. It contains many items of professional interest.

The *Wisconsin Medical Recorder* is the title of a journal that appeared at the beginning of the year and is published at Janesville. It is edited by Dr. J. P. Thorne and is published by Hall and Thorne. The profession of Wisconsin deserves a first-class medical periodical.

The editorial and publication offices of the *North Carolina Medical Journal* have been removed from Wilmington to Winston, N. C.

*Mathews's Medical Quarterly*, now in the fifth year of its publication, announces that it will be printed hereafter as a monthly with a change in name to the *Louisville Journal of Surgery and Medicine.* Wisdom and prosperity are both indicated in this announcement: wisdom, because personal names of journals are not desirable; and prosperity, because a change from a quarterly to a monthly indicates professional strength and financial solidity.

---

*Polk's Medical and Surgical Register of the United States and Canada* is now undergoing its fifth revision, and physicians who have not given their names to the canvassers are urged to send them to the publishers at once. Address R. L. Polk & Co., Detroit, Mich.

